# Evaluation of a Novel “Quad” Wavelength Light Curing Unit

**DOI:** 10.4317/jced.59825

**Published:** 2022-10-01

**Authors:** Kevin R. Adams, Daniel A. Savett, Wen Lien, Christopher Raimondi, Kraig S. Vandewalle

**Affiliations:** 1 DDS. Maj, USAF, DC. Resident. Advanced Education in General Dentistry Residency. AF Postgraduate Dental School. 1615 Truemper St. Joint Base San Antonio - Lackland, TX, USA, 78236. Uniformed Services University of the Health Sciences, Bethesda, MD, USA; 2 Col (ret), USAF, DC. Dental Service Point of Contact. Contractor, Axiom Resource. Management, Inc.Dental Program Section - Purchased Care Delivery Branch. TRICARE Health Plan Division. Defense Health Agency (DHA). 2941 Fairview Park Dr # 850, Falls Church, VA, 22042, USA; 3 DMD, MS, MS. Col, USAF, DC. Director of Dental Materials Research. USAF Dental Research and Consultation Service. 3650 Chambers Pass, Bldg 3610. Joint Base San Antonio - Fort Sam Houston, TX, USA, 78234. Uniformed Services University of the Health Sciences, Bethesda, MD, 20814, USA; 4 DDS, MS, MS. Lt Col, USAF, DC. Co-Director of Dental Materials Research. USAF Dental Research and Consultation Service. 3650 Chambers Pass, Bldg 3610. Joint Base San Antonio - Fort Sam Houston, TX, USA 78234. Uniformed Services University of the Health Sciences, Bethesda, MD, USA; 5 DDS, MS. Col (ret), USAF, DC. Director of Dental Research. Advanced Education in General Dentistry Residency. AF Postgraduate Dental School. 1615 Truemper St. Joint Base San Antonio - Lackland, TX, USA 78236. Uniformed Services University of the Health Sciences, Bethesda, MD, USA

## Abstract

**Background:**

This study investigated the properties (depth of cure, surface hardness, and volumetric shrinkage) of two composite restorative materials when polymerized with a novel “quad” spectrum (PinkWave) light-curing unit (LCU) compared to a tri-spectrum LCU (Valo Grand).

**Material and Methods:**

One Valo Grand LCU was modified to be similar in irradiance to the PinkWave, and a second Valo Grand was utilized at the manufacturer’s standard irradiant settings. Depth of cure was evaluated using the scraping technique (ISO 4049). Top and bottom surface hardness and bottom/maximum hardness ratios were determined using a hardness tester. Volumetric shrinkage was determined using a video-imaging device. Additionally, the surface temperature of the light tips of the LCUs was measured using a K-type thermocouple.

**Results:**

No significant difference in depth of cure was found with either composite between the PinkWave LCU and the modified Valo Grand LCU at similar irradiance. The unadjusted Valo Grand LCU had slightly less depth of cure. There was no difference in top or bottom surface hardness, bottom/maximum hardness ratios, or volumetric shrinkage between any of the LCU curing modes per composite type. The PinkWave LCU had a significantly greater increase in heat at the tip compared to the modified Valo Grand LCU at similar irradiance and the unadjusted Valo Grand LCU.

**Conclusions:**

The new quad-spectrum LCU, PinkWave, had a significant increase in surface temperature without any improvement in the composite properties tested compared to the tri-spectrum LCU, Valo Grand, at similar irradiance.

** Key words:**Light-curing unit, emission spectrum, composite resin, mechanical and physical properties.

## Introduction

The clinical performance of dental composite restorative materials is dependent on many factors. One fundamental factor for contributing to a successful restoration is the proper selection and application of a light-curing unit (LCU). Often overlooked, LCUs can have an effect on depth of cure, degree of conversion, hardness, and polymerization kinetics of composites (1). If a composite does not receive adequate energy from an LCU, or if the wavelength of the light does not activate the specific photoinitiator in the composite, its polymerization will be reduced (2). When a composite is not properly polymerized, its mechanical strength and marginal integrity are significantly decreased, and water sorption is significantly increased (3-5). An arbitrary increase of the curing time to prevent the under-curing of a composite can increase the temperature of the tooth, potentially damaging the pulp and surrounding tissues (6). The ultimate goal is to improve the mechanical properties of the restoration while minimizing heat transfer to the tooth.

 Another key component of successful polymerization is to ensure the spectrum of light delivered by the LCU coincides with the photonic absorption of the photoinitiator in the composite (7). Light-emitting diode (LED) LCUs with a single-spectral emission in the blue wavelength were originally introduced as an alternative to quartz-tungsten-halogen (QTH) LCUs with the benefits that they can be more physically compact, energy-efficient, cordless, and do not require bandpass filters to isolate blue light (8). Single-spectrum LED LCU emission is relatively more narrow and often centered near 470 nm to match the absorbance range of camphorquinone (CQ), which lies between 400-500 nm with a peak sensitivity at 468 nm (9). Having a narrow spectrum tailored to the target photoinitiator is ideal because most of the energy delivered is able to be quantized to generate free radical formation, and less is converted into molecular kinetic energy (heat). Some manufacturers use other photoinitiators, such as trimethylbenzoyl-diphenylphosphine oxide (TPO), which is less yellow in color, to create whiter restorative shades. These alternative initiators are usually sensitive to ultraviolet or violet light or a wavelength between 380 and 410 nm (9). Products containing these alternative photoinitiators may not be effectively polymerized by single-spectrum LCUs that emit light at 400-500 nm wavelengths (10). In response, some manufacturers market LCUs with additional LED diodes to deliver multiple different spectral emission peaks (multi-spectra) that correspond to the various absorption spectra of the different photoinitiators. A multi-spectral LED LCU with emission peaks near 380-410 nm and 470 nm is generally ideal to effectively polymerize the wide range of dental adhesives and composites available today (11).

Vista-Apex Dental (Racine, WI, USA) recently introduced a novel LCU called the *Pi*nkWave that reportedly provides “pink light” instead of the traditional “blue light.” Vista-Apex Dental claims the PinkWave has a patented “Quad Wave” technology with spectral emission peaks at 410nm (violet), 470nm (blue), 625nm (red), and 840nm (near-infrared). Due to these additional wavelengths, the manufacturer states the PinkWave LCU reduces polymerization shrinkage of composites by 37%, increases polymerization by 23%, and reduces energy absorption from the pulp. The PinkWave has reported irradiances of 1515 or 1720 mW/cm2 depending on mode (12). No research has been published evaluating the novel PinkWave LCU.

The purpose of this study was to compare the depth of cure, top and bottom surface hardness, bottom/maximum hardness ratios, volumetric polymerization shrinkage, and the surface temperature at the LCU tip using two composite restorative materials (Esthet-X, Dentsply Caulk, Milford, DE, USA; Tetric EvoCeram, Ivoclar Vivadent, Schaan, Liechtenstein) polymerized with the novel PinkWave and the Valo Grand (Valo Grand, Ultradent Products, South Jordan, UT, USA) LCUs. These observations allowed the examination of the polymerization efficacy of each LCU. The Valo Grand LCU has a similar tip surface area (107 mm2) compared to the PinkWave LCU (113 mm2) (12). The Valo Grand is a tri-spectrum LED LCU with emission peaks near 405, 445, and 465 nms and irradiances of 1000, 1600, or 3200 mW/cm2, depending on the mode (13). The PinkWave LCU was compared to an adjusted Valo Grand LCU with similar irradiance and to another Valo Grand LCU with no adjustments to the irradiance. Esthet-X HD and Tetric EvoCeram were chosen as composite resins because Esthet-X HD has just one photoinitiator (CQ), while Tetric EvoCeram has two photoinitiators (TPO and CQ).

The null hypotheses were that there would be no differences in the depth of cure, top and bottom surface hardness, bottom/maximum hardness ratios, volumetric polymerization shrinkage, or the surface temperature of the LCU tip when two common dental composite resins were polymerized by the PinkWave LCU, an adjusted Valo Grand LCU at similar irradiance to the PinkWave LCU, or an unadjusted Valo Grand LCU.

## Material and Methods

The LCUs were used in standard mode with a 10-second exposure time. One Valo Grand was modified to emit a similar irradiance to the PinkWave LCU to reduce the variability associated with unequal irradiances. A second unmodified Valo Grand LCU was used as a control. See Tables  Table 1 and  Table 2 below for more details on the composites and LCUs utilized in this study.

**Table 1:**
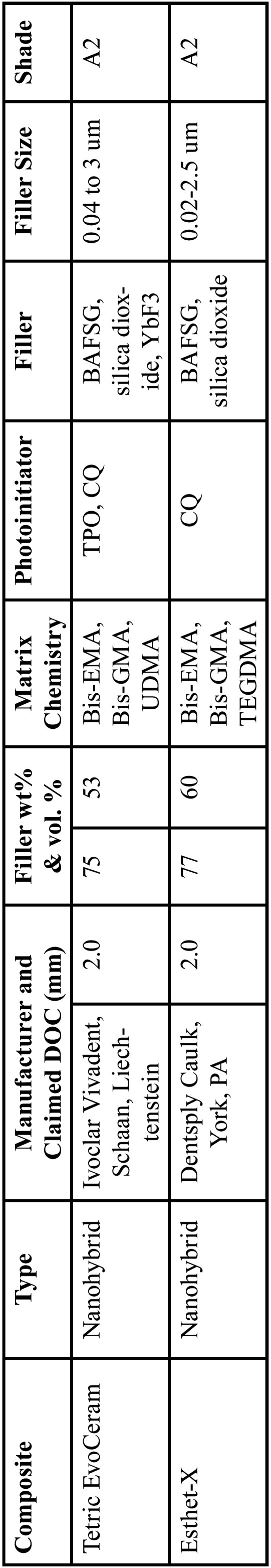
Components of the composite restorative materials.

**Table 2:**
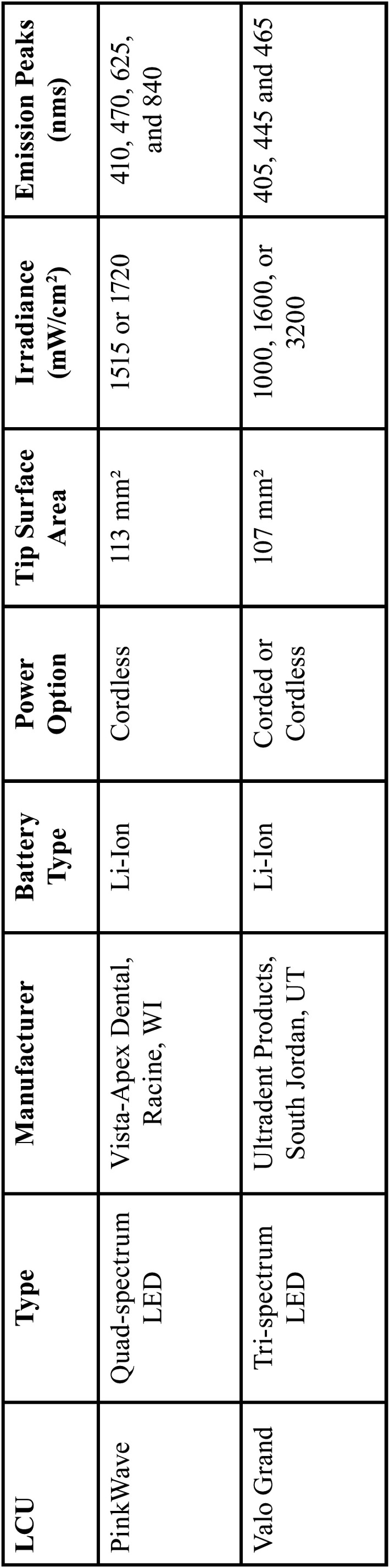
Manufacturer reported specifications of the light-curing units.

-Modification of the Power

**Figure 1:**
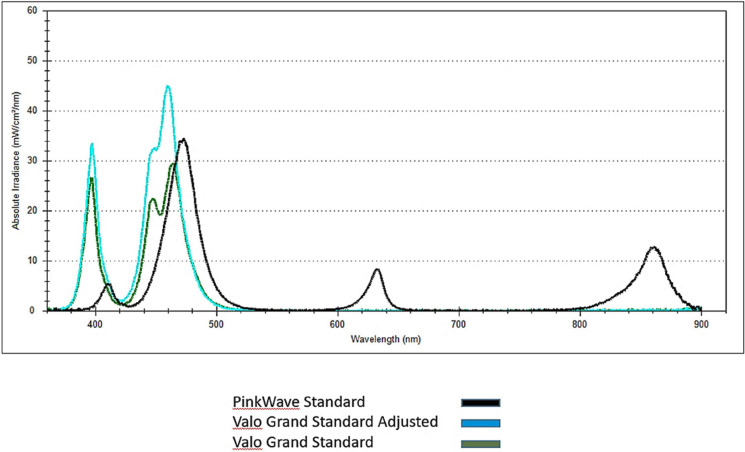
Emission spectrums of the LCUs.

The radiant power of the Valo Grand LCU was modified by adjusting the applied voltage to the LED assembly to be similar to the power of the PinkWave LCU. A regulated, adjustable, constant voltage DC power supply source (Eventek KPS305D, Shen Zhen Sheng Ya Hardware Products Co., Ltd, China) was supplied to the LED assembly by first passing through an electrically isolated interval timer circuit. Briefly, a separately powered, voltage regulated, monostable multivibrator circuit produced a timed, one-shot square wave output. This square wave duration was determined partially by using fixed resistors within the circuit but also a variable resistor for fine adjustment. This output turned on and off a power metal-oxide-semiconductor field-effect transistor (MOSFET) that in turn energized a mechanical relay that controlled the applied voltage from the Eventek DC power source to the LED assembly. Thus, the timing circuit was electrically isolated from the LED irradiance voltage in both its operation as a one-shot timer and as an activator of the LED assembly. A manual override was made to allow adjustment and measurement of the irradiance by the operator before utilizing the timed protocol. Through this method, an applied voltage to the irradiance standard curve was established. The irradiance and emission spectrums of both LCUs were measured using the 4mm diameter sensor of a spectrophotometer (MARC Light Collector, Blue Light Analytics, Halifax, Canada). The irradiance of each LCU was measured ten times, and a mean and standard deviation was determined, (Fig. 1, Table 3).

**Table 3:**
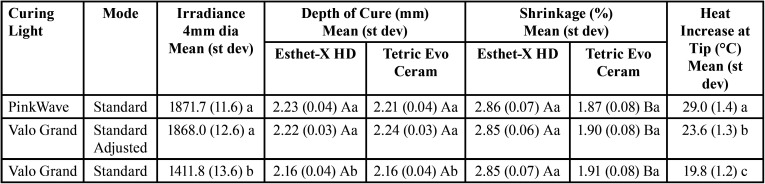
Irradiance, depth of cure, shrinkage of the composites, and heat increase at the tip using the LCUs.

-Depth of Cure

To determine the depth of cure, ten specimens of each composite were tested using the scraping technique (ISO Standard 4049) (14). A 4 mm diameter by 8 mm long stainless-steel split mold (Sabri Dental Enterprises, Downers Grove, IL, USA) was placed on a plastic-strip-covered glass slide on a standard white background. The composite was injected into the mold, a plastic strip was placed, and the composite was condensed with a glass slide to displace excess resin. The glass slide was removed, and the composite was immediately polymerized for 10 seconds with the LCUs. The LCUs were positioned with a clamp so that they were flush with the top surface of the plastic-strip-covered composite. The uncured resin was then scraped with a plastic instrument starting from the deepest point on the underside of the mold until the polymerized resin was reached. The specimens were visually inspected and discarded if any voids were noted. The length of the remaining polymerized material was measured with an electronic digital caliper (GA182, Grobet Vigor, Carlstadt, NJ, USA) and divided by two according to the ISO standard (14). A mean depth of cure (mm) and standard deviation were determined for each material and LCU curing mode.

-Surface Hardness

Sixty composite specimens (30 Tetric EvoCeram, 30 Esthet-X HD) were created to evaluate surface hardness. A cylindrical plastic split mold, 2.0 mm in height and 8.0 mm in diameter (Sabri Dental Enterprises), was placed on a plastic-strip-covered glass slide on a standard white background as before. The composite was inserted into the mold. Another plastic strip was then placed on top, while a microscope glass slide was used to flatten the top surface. Afterward, the glass slide was removed. The LCUs were positioned with a clamp as before. The composites were light-cured for 10 seconds. Following light curing, specimens were stored in the dark at 37°C in 100% humidity for 24 hours in an incubator (Model 20 GC, Quincy Lab Corp, Chicago, IL, USA). Three hardness indentations were made on the top and the bottom of each specimen in the central 4 mm area of the specimen surface using a load of 100 grams for ten seconds in a Knoop hardness testing device (LECO, LM-300AT, St. Joseph, MI, USA). The mean top and bottom Knoop hardness value and standard deviation were determined for each material and LCU curing mode. In addition, the percent bottom/maximum Knoop hardness ratio was calculated by dividing the bottom surface hardness by the maximum recorded hardness per material and multiplying by 100.

-Percent Volumetric Polymerization Shrinkage

To determine volumetric polymerization shrinkage, 4mm-diameter composite specimens were placed on a pedestal in a video-imaging device (AcuVol, Bisco, Schaumberg, IL, USA). Ten specimens of each composite per LCU were imaged from the side at a distance of 10cm. The video camera digitized and analyzed the images with the provided image-processing software. The specimens were cured separately with each LCU mode for 10 seconds of curing time. Percent polymerization shrinkage was recorded continuously for 10 minutes after the light initiation. A mean percent volumetric polymerization shrinkage and standard deviation were determined for each material and LCU curing mode.

-Surface Temperature of LCU Tip

To measure the surface temperature of the LCU tip, a K-type thermocouple wire probe (Digi-Sense Type-K Wire Probes, 30 Gauge; Cole-Parmer, Vernon Hills, IL, USA) was connected to a data-logging thermometer (SDL200 4-Channel Datalogging Thermometer, Extech, Nashua, NH, USA) and positioned in contact with the center of the LCU tip. A baseline temperature was recorded. The maximum increase in temperature was recorded after a 10-second exposure time. Ten temperature readings were recorded (8). A mean increase in surface temperature and standard deviation were determined for each LCU curing mode.

The data were analyzed with two-way and one-way ANOVAs with Tukey’s post hoc tests and unpaired t-tests (alpha=0.05) ( Table 3, Table 4).

**Table 4:**
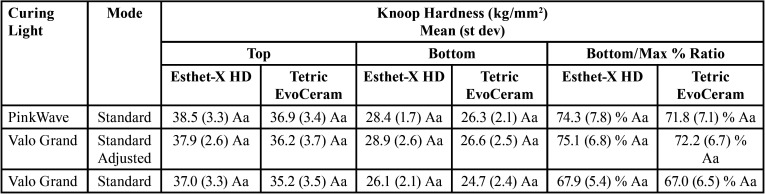
Top and bottom surface hardness and bottom/maximum hardness ratios of the composites.

## Results

The mean irradiance of the PinkWave LCU was found to be 1871.7±11.6 mW/cm2 in standard mode, which was not significantly different (*p*=0.93) from the modified Valo Grand LCU at 1868.0±12.6 mW/cm2 in standard mode. The irradiance of the PinkWave LCU and adjusted Valo Grand LCU were significantly greater (*p*<0.001) than the unadjusted Valo Grand LCU in standard mode (1411.8±13.6 mW/cm2).

 The depth of cure of the composite materials using the PinkWave LCU was not significantly different from the adjusted Valo Grand LCU (*p*=0.77), but both were greater than the unadjusted Valo Grand LCU (*p*<0.001). There was no significant difference in depth of cure between the two composite types (*p*>0.10). Additionally, there was no significant difference in surface hardness based on the type of composite (*p*>0.08) or LCU curing mode (*p*>0.07) for both the top and bottom surfaces. When evaluating the bottom/maximum hardness ratios, no significant difference was found in percent hardness ratios based on composite type (*p*>0.49) or LCU curing mode (*p*>0.31). The percent volumetric shrinkage of the composites was not significantly different between the various LCU curing modes (*p*>0.61), but Tetric Evo Ceram had significantly lower shrinkage (*p*<0.001) than Esthet-X HD. The PinkWave LCU had the greatest increase in temperature at the tip (29.0±1.4°C), which was significantly greater (*p*<0.001) than the adjusted Valo Grand LCU (23.6±1.3°C). The unadjusted Valo Grand LCU had the lowest increase in temperature at the tip (19.8±1.2°C) and was significantly less (*p*<0.01) than the PinkWave LCU and adjusted Valo Grand LCU.

## Discussion

This study evaluated the quad-spectrum PinkWave, a novel LCU that contains emission peaks in the red and near-infrared in addition to the more traditional peaks in the violet and blue wavelengths of light. With the additional emission at higher wavelengths, the manufacturer claims that the PinkWave LCU reduces shrinkage and increases polymerization while reducing the energy absorption by the pulp. The tri-spectrum Valo Grand LCU, with emission peaks in the violet and blue wavelengths of light, was selected as a control to compare the efficacy of the additional red and infrared wavelengths of PinkWave LCU.

To reduce variability while testing the LCUs, the LCU handpieces were stabilized with a clamp to align and center the light tip during testing. Also, all tests were conducted with 10 seconds of exposure time from the LCUs. Research has demonstrated that the emitted light from LCUs may not be uniform across the active diameter of the light beam (15). In order to standardize the radiant exposure to the composite resin specimens, the power was measured in the center 4 mm of the light tip of the LCUs using a spectrophotometer with a 4 mm diameter sensor to match the 4 mm diameter of the mold used for the depth of cure test (ISO Standard 4049). Surface hardness measurements were made in the center 4 mm of the composite specimens, and 4 mm diameter composite specimens were created and light-cured when evaluating polymerization shrinkage. Additionally, the surface temperature was measured in the center of the light guide with the thermocouple. Two nanohybrid composites containing different combinations of photoinitiators were selected to evaluate the potential effect of the various emission wavelengths of the LCUs on the properties of the composites.

Depth of cure and surface hardness were selected to examine the polymerization of the composite specimens. Significant differences were found in the depth of cure based on the type of LCU curing mode, so the null hypothesis was rejected. Depth of cure was not significantly different between the PinkWave LCU and the adjusted Valo Grand LCU with both composite types. The depth of cure of the composites was significantly lower with the unadjusted Valo Grand LCU with lower irradiance, but it was only a difference of 0.06 mm for the Esthet-X HD and 0.08 mm for Tetric EvoCeram, both of which may not be clinically significant. The low variability of the scrape test for determining the depth of cure could have contributed to the statistical differences between the groups. The null hypothesis was not rejected for surface hardness. The top and bottom surface hardness values and hardness ratios were not significantly different between the PinkWave LCU and the adjusted or unadjusted Valo Grand LCU with both composite types. Although there was a trend with the unadjusted Valo Grand LCU with lower irradiance to demonstrate lower surface hardness and hardness ratios, the differences were not statistically significant.

Figure 1 displays the emission spectrum of the PinkWave LCU and the adjusted and unadjusted Valo Grand LCUs. Although the overall irradiance of the adjusted Valo Grand LCU was similar to the *Pi*nkWave LCU, the absolute irradiance (mW/cm2/nm) was greater for the adjusted Valo Grand LCU in the violet and blue wavelengths than the PinkWave LCU and the unadjusted Valo Grand LCU. In spite of the differences in absolute irradiances between the PinkWave LCU and the adjusted Valo Grand LCU at these wavelengths, no difference in depth of cure and surface hardness was demonstrated between the two LCUs per composite. Additionally, there was no difference in depth of cure or surface hardness between the two composite types. Esthet-X HD only contains CQ, and Tetric EvoCeram contains both CQ and TPO. Although the Valo Grand LCUs had relatively higher levels of absolute irradiance in the violet spectrum compared to the PinkWave LCU, both LCUs emit wavelengths in both the violet and blue spectrums to polymerize both CQ and TPO. However, wavelengths in the red and infrared regions were detectable for the PinkWave LCU and not the Valo Grand LCUs. The authors are not aware of any commercially available dental restorative composites with photoinitiators sensitive to light in the red or near-infrared range (16).

Esthet-X HD had significantly greater volumetric polymerization shrinkage compared to Tetric EvoCeram, regardless of LCU curing mode. However, there was no significant difference in polymerization shrinkage per composite type based on LCU curing mode, and therefore, the null hypothesis was not rejected. The PinkWave LCU did not provide a decrease in polymerization shrinkage compared to the Valo Grand LCUs. The PinkWave LCU, however, had a significant increase in heat at the light tip compared to the adjusted or non-adjusted Valo Grand LCUs. The manufacturer, however, claims that the PinkWave LCU reduces the energy absorption from the pulp (12). Although this study did not measure pulpal temperatures, *in vivo* studies have demonstrated a relationship between the irradiance of LCUs and pulpal temperature (17,18). Other than the undesirable production of greater surface heat at the light tip, the emission spectrum of the light from the PinkWave LCU in the red or near-infrared wavelengths did not contribute to an increase in surface hardness, which indirectly measures the degree of polymerization, or to a reduction of polymerization shrinkage of the composite specimens tested in this study.

## Conclusions

The novel quad-spectrum LCU, PinkWave, had a significant increase in surface temperature without any improvement in polymerization efficacy compared to the tri-spectrum LCU, Valo Grand, at similar irradiance.

## References

[1] Santini A, Turner S (2011). General dental practitioners' knowledge of polymerisation of resin-based composite restorations and light curing unit technology. Br Dent J.

[2] Halvorson RH, Erickson RL, Davidson CL (2002). Energy dependent polymerization of resin-based composite. Dent Mater.

[3] Price RB, Ferracane JL, Shortall AC (2015). Light-curing units: a review of what we need to know. J Dent Res.

[4] Rueggeberg FA (2011). State-of-the-art: dental photocuring-a review. Dent Mater.

[5] Peutzfeldt A, Asmussen E (2005). Resin composite properties and energy density of light cure. J Dent Res.

[6] Price RB, Shortall AC, Palin WM (2014). Contemporary issues in light curing. Oper Dent.

[7] Stansbury JW (2000). Curing dental resins and composites by photopolymerization. J Esthet Dent.

[8] Gomes M, DeVito-Moraes A, Francci C, Moraes R, Pereira T, Froes-Salgado N (2013). Temperature increase at the light guide tip of 15 contemporary LED units and thermal variation at the pulpal floor of cavities: an infrared thermographic analysis. Oper Dent.

[9] Shimokawa C, Sullivan B, Turbino ML, Soares CJ, Price RB (2017). Influence of emission spectrum and irradiance on light curing of resin-based composites. Oper Dent.

[10] Santini A, Gallegos IT, Felix CM (2013). Photoinitiators in dentistry: a review. Prim Dent J.

[11] Ilie N, Hickel R (2008). Can CQ be completely replaced by alternative initiators in dental adhesives?. Dent Mater J.

[12] (2020). Pink Wave Pink Differently. https://vistaapex.com/pinkwave.

[13] (2020). Valo Grand. https://www.ultradent.com/products/categories/equipment/curing-lights/valo-grand.

[14] (2019). Dentistry Polymer-based filling, Restorative Materials.

[15] Price RB, Ferracane JL, Hickel R, Sullivan B (2020). The light-curing unit: an essential piece of dental equipment. Int Dent J.

[16] Kowalska A, Sokolowski J, Bociong K (2021). The photoinitiators esed in resin based dental composite - a review and future perspectives. Polymers (Basel).

[17] Zarpellon DC, Runnacles P, Maucoski C, Gross DJ, Coelho U, Rueggeberg FA (2018). Influence of Class V preparation on in vivo temperature rise in anesthetized human pulp during exposure to a Polywave® LED light curing unit. Dent Mater.

[18] Runnacles P, Arrais CA, Pochapski MT, Dos Santos FA, Coelho U, Gomes JC (2015). In vivo temperature rise in anesthetized human pulp during exposure to a polywave LED light curing unit. Dent Mater.

